# Changes of insulin receptors in high fat and high glucose diet mice with insulin resistance

**DOI:** 10.1080/21623945.2023.2264444

**Published:** 2023-10-13

**Authors:** Chen Lei, Jing Wang, Xin Li, Yuan-Yuan Mao, Jian-Qun Yan

**Affiliations:** a Physiological Department, Xi’an Jiaotong University College of Medicine, Xi’an, China; b Department of geriatrics and special needs, General Hospital of Ningxia Medical University, Yinchuan, China; c Research Office, General Hospital of Ningxia Medical University, Yinchuan, China; d Department of nutrition, General Hospital of Ningxia Medical University, Yinchuan, China

**Keywords:** insulin resistance, liver, ovary, insulin signalling, skeletal muscle

## Abstract

This study aimed to observe the expression of insulin-signaling molecules in different organs of mice with insulin resistance (IR). Firstly, mice were fed a high-fat and high-sugar diet (HF group) to establish an IR model, and the controls (NF group) were fed with a normal diet. Next, the weight, fasting blood glucose (FBG), serum insulin and insulin tolerance were detected. Pathological changes of liver tissues were observed by H&E staining. The expressions of INSR, IRS-1 and IRS-2 in the liver, skeletal muscle and ovary were measured by qRT-PCR and western blotting. As a result, compared with the NF group, the HF group mice had increased weight, FBG, insulin and IR index after 6-week of feeding as well as a worse performance in the insulin tolerance test and H&E staining showed fatty liver-like changes after 12-week of feeding, exhibited lower expression of INSR, IRS-1 and IRS-2 in the liver of mice at 6 and 12 weeks. The expression of INSR and IRS-1 in skeletal muscle tissues exhibited the same trend, while those in ovary organs showed the opposite trend. These results suggested that the insulin signaling alters in the liver, skeletal muscle and ovary organs with the progress of IR.

## Introduction

The continuous improvement in living standards has changed the dietary habits of people, and the changed dietary habits are associated with gradually increasing insulin resistance. During insulin resistance, a great deal of insulin secreted by the body compensates for the glucose metabolism disorder, thereby gradually developing into hyperinsulinemia [1]. Insulin resistance is the ‘common soil’ of endocrine and metabolic diseases. The liver, as the target tissue of insulin [2], is related to the inhibition of gluconeogenesis and the activation of adipogenesis [3]. Hepatic insulin resistance refers to a decrease in the sensitivity of the liver to insulin, causing gluconeogenesis and hyperglycaemia [4–7]. It was reported that an increase in plasma insulin concentration stimulated glucose uptake in skeletal muscle [8]. When the primary defect is located in skeletal muscle, remediating insulin resistance in the muscle alone is sufficient to restore whole-body glucose homoeostasis [9]. Polycystic ovary syndrome (PCOS), characterized by excessive androgen and chronic anovulation, is a common endocrine disorder in premenopausal women [10,11]. Moreover, the pathogenesis of PCOS is complicated.

In 1980, Burgen et al. reported that PCOS patients were referred to populations with insulin resistance and hyperinsulinemia [12,13] and that hyperinsulinemia would lead to a decrease in insulin clearance and an increase in insulin secretion [14]. Insulin receptor (INSR) protein, as an adaptor protein, regulates biological processes such as cell growth, metabolism, survival and proliferation by binding to transmembrane receptors and coordinating cell signal transmission from extracellular to intracellular [15]. Mutations of specific tyrosine residues in INSR proteins can seriously impair the ability of insulin to stimulate glycogen synthesis, glucose uptake and oxidation, insulin growth-promoting effects, and induce other acute metabolic diseases [16]. Although numerous studies on insulin resistance in the liver, skeletal muscle and ovaries have been published, most have only focused on one organ. Hence, an in-depth understanding of the changes of INSRs in different organs is of great significance to developing novel therapeutic options for patients with hyperinsulinemia.

Therefore, an insulin resistance mouse model was established in this study to assess the expression levels of several related biomarkers, i.e. INSR, insulin receptor substrate-1 (IRS-1) and phospho-IRS-1 (p-IRS-1), in the liver, skeletal muscle and ovary at different time points to understand the insulin signalling changes in different organs under insulin resistance.

## Results

### Physical and organ changes of mice in each group

**Figure 1.**
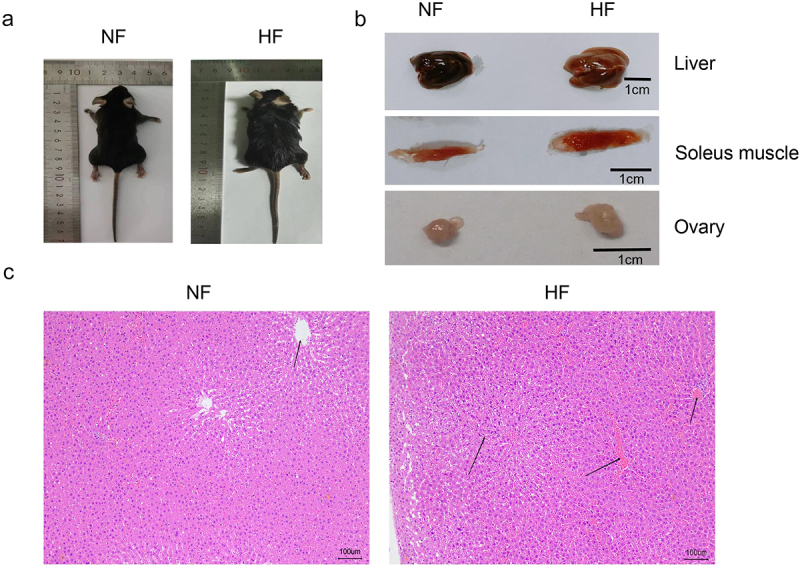
Body and organ changes of mice in the two groups after 12-week feeding. a: images of extrinsic feature of mice in the two groups after 12-week feeding. b: images of extrinsic feature of liver, soleus muscle and ovary organs of mice in the two groups after 12-week feeding. c: H&E staining of liver tissue of mice in the two groups after 12-week feeding. NF group, normal diet group; HF group, high-fat and high-sugar diet group.

Based on the physical appearance, the physique, the volume of the liver, soleus muscle and ovary of mice in the HF group was significantly greater than those of mice in the NF group (Figure 1a-b). The H&E staining results showed fatty liver-like changes with intracellular fat vacuoles in the liver tissues of mice in the HF group (Figure 1c).

## Changes in body weight, fasting blood glucose and serum insulin of mice in the two groups of mice in different periods

**Figure 2.**
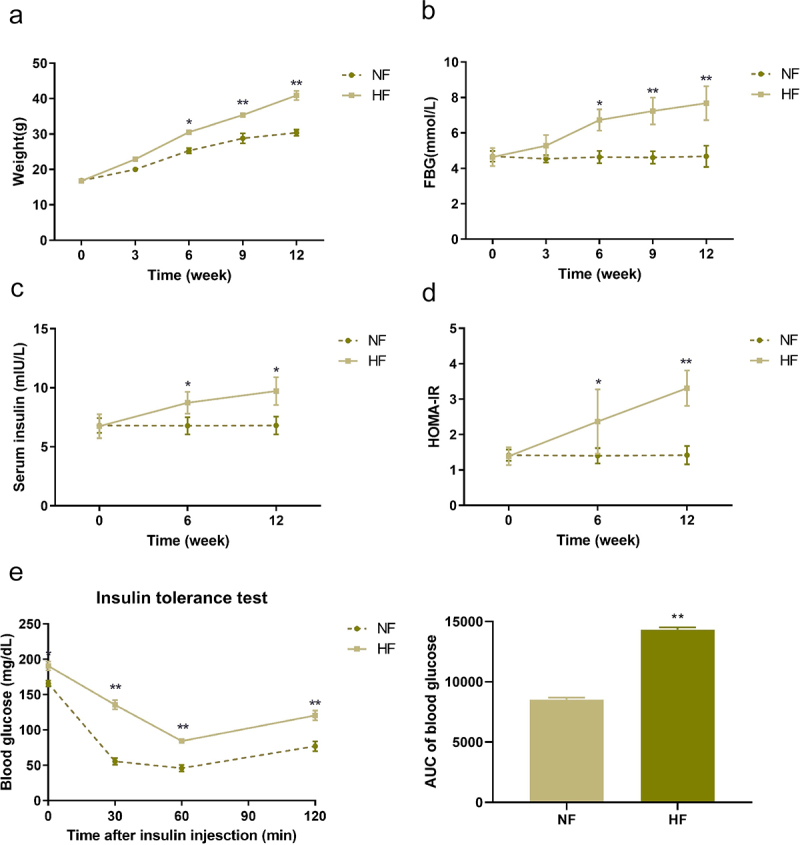
Changes of weight, FBG and serum insulin in two groups of mice in different periods. a: changes of weight in two groups of mice in different periods. b: changes of FBG levels in two groups of mice in different periods. c: changes of serum insulin in two groups of mice in different periods. d: changes of HOMA-IR in two groups of mice in different periods. e: insulin tolerance test in two groups of mice after 12 weeks of feeding. *P < 0.05, **P < 0.01 *vs*. NF group.

There was no significant difference in body weight, fasting blood glucose (FBG) and serum insulin between the NF group and HF group before modelling. After feeding for 6 weeks, the body weight and FBG of mice in the HF group were significantly increased compared with those of mice in the NF group (*P* < 0.05), and the difference increased with time (Figure 2a-b). In the 9th and 12th weeks, mice in the HF group presented with notably higher serum insulin and insulin resistance indexes than mice in the NF group (*P* < 0.05) (Figure 2c-d). Besides, the insulin tolerance test was detected after 12 weeks of feeding. The results showed that after injection of insulin, the rate of decline of blood glucose levels of the HF group were lower than that in the NF group, and the AUC of glucose in the HF group were significantly increase compared to the NF group (*P* < 0.05) (Figure 2e). These results indicated that we successfully established the insulin resistance mice models.

### Changes of insulin receptor-related mRNA expression levels in different periods

**Figure 3.**
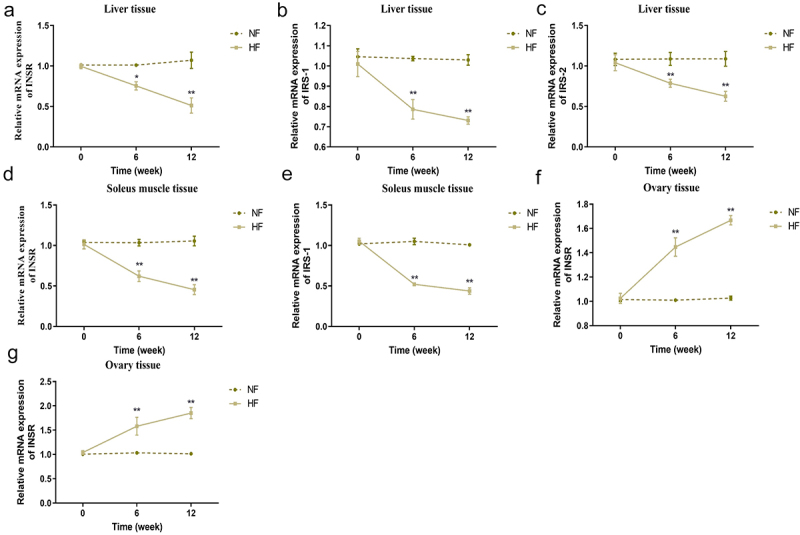
Changes of insulin receptor-related mRNA expression levels in different periods. a – c: changes in INSR mRNA (a), IRS-1 mRNA (b) and IRS-2 mRNA (c) in the liver tissues of mice in the two groups at different time points. d-e: changes in INSR mRNA (d) and IRS-1 mRNA (e) in the soleus muscle tissues of mice in the two groups at different time periods. f-g: changes in INSR mRNA (f) and IRS-1 mRNA (g) in the ovary tissues of mice in the two groups at different time periods. *P < 0.05, **P < 0.01 *vs*. NF group.

There was no significant difference in the expression of INSR, IRS-1 and IRS-2 mRNA in the liver, soleus muscle and ovary tissues between the two groups before modelling. The expression of INSR, IRS-1 and IRS-2 mRNA in the liver of mice in the HF group was gradually lower than that in the NF group from 6 weeks to 12 weeks (*P* < 0.05; Figure 3a-c). The expression of INSR and IRS-1 in soleus muscle tissues of mice between the two groups exhibited the same trend with the liver tissues (*P* < 0.05; Figure 3d-e). Nevertheless, the expression levels of INSR and IRS-1 mRNA in the ovary tissues of mice in the HF group showed the opposite trend, namely, both expression levels increased gradually and were higher than those in the NF group from 6 to 12 weeks (*P* < 0.05; Figure 3f-g).

### Changes in the expression of insulin receptor-related proteins in the liver During different periods

**Figure 4.**
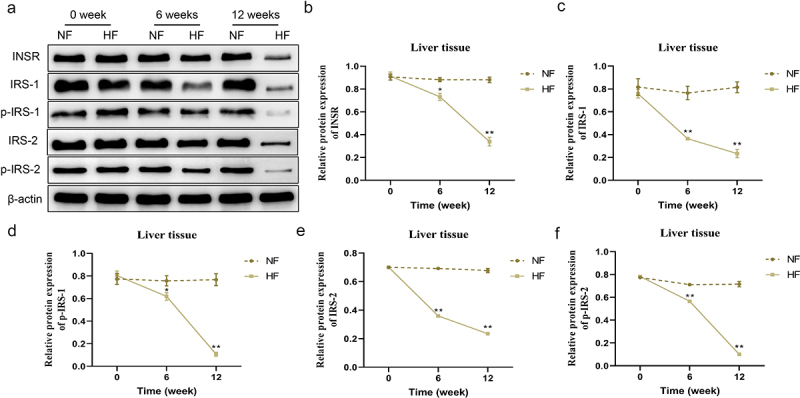
Changes of insulin receptor-related proteins expression levels in the liver tissues in different periods. a – f: changes in the protein expression levels of INSR, IRS-1, p-IRS-1, IRS-2 and p-IRS-2 in the liver tissues of mice in the two groups at different periods. *P < 0.05, **P < 0.01 *vs*. NF group.

**Figure 5.**
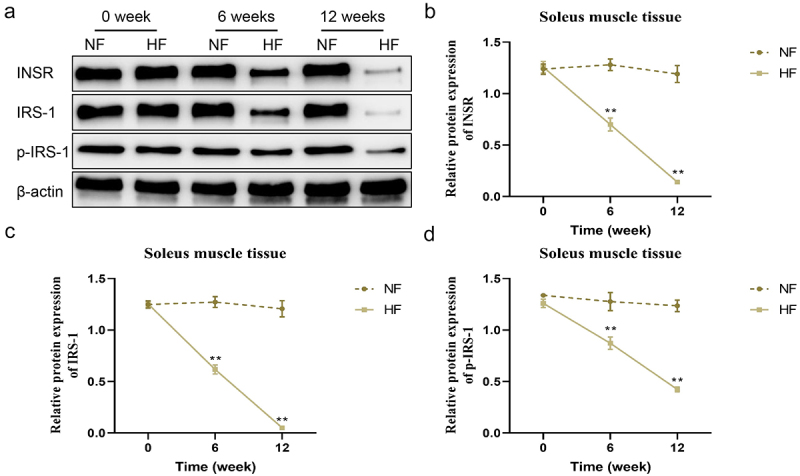
Changes of insulin receptor-related protein expression levels in the soleus muscle tissues in different periods. a – d: changes in the protein expression levels of INSR, IRS-1 and p-IRS-1 in the soleus muscle tissues of mice in the two groups at different periods. **P < 0.01 *vs*. NF group.

**Figure 6.**
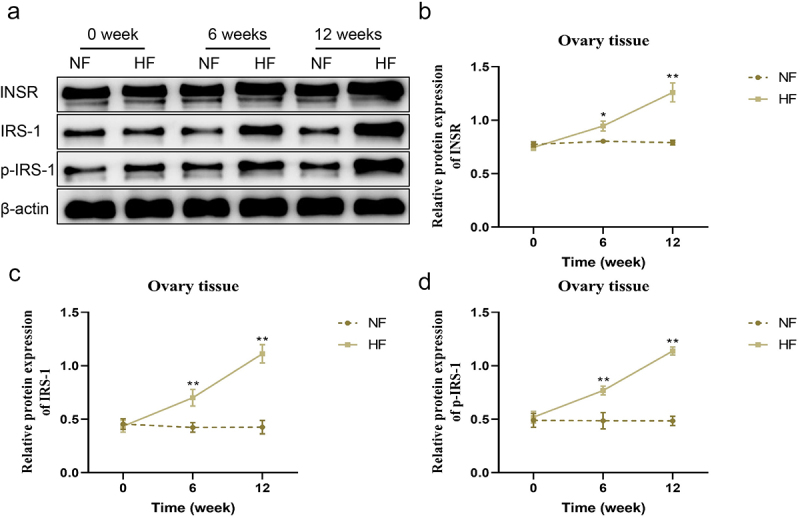
Changes of insulin receptor-related protein expression levels in the ovary tissues in different periods. a – b: changes in the expression levels of INSR, IRS-1 and p-IRS-1 in the ovary tissues of mice in the two groups at different periods. *P < 0.05, **P < 0.01 *vs*. NF group.

The expression levels of INSR, IRS-1, p-IRS-1, IRS-2 and p-IRS-2 proteins in the liver, soleus muscle and ovary tissues were not significantly different between the two groups before modelling. In contrast to the NF group, the expression of INSR, IRS-1, p-IRS-1, IRS-2 and p-IRS-2 proteins in the liver of mice in the HF group was gradually lower from 6 weeks to 12 weeks (*P* < 0.05; Figure 4a-f). The expression of INSR, IRS-1 and p-IRS-1 proteins in soleus muscle tissues of mice between the two groups exhibited the same trend with the liver tissues (*P* < 0.05; Figure 5a-d). However, the expression of INSR, IRS-1 and p-IRS-1 proteins in the ovary tissues of mice in the HF group showed the opposite trend, which were gradually increased and higher than in the NF group from 6 weeks to 12 weeks (*P* < 0.05; Figure 6a-d).

## Discussion

The high-fat and high-sugar diet used in this study was improved according to the previous relevant literature, and the improved diet could fully induce abnormal insulin secretion of target organs and gradually result in insulin resistance [17]. At present, researchers believe that increased insulin secretion usually occurs before insulin resistance and play an important role in the progression of diabetes. Based on the experimental results in this study, the weight of mice in each group did not excessively increase within 12 weeks. However, the whole experimental period was relatively short, and the liver and abdominal fat of mice in the HF group increased significantly in each period. Thus, the success rate of modelling may be determined by the comprehensive biochemical indicators related to glucose metabolism and lipid metabolism, rather than just dependent on the weight increase of mice in each period.

The indicators used in this study showed that the results of mice in the HF group were more significant than mice in the NF group. Although the statistical results between the NF group and HF group in the sixth week were around the critical value of 0.05, the comparison difference between the two groups in the 12th week was still very obvious. Such a result indicated that the insulin at this time could not fully exert its normal physiological and metabolic function. Moreover, the body had already been considered to manifest early insulin resistance by this time. Due to insulin resistance, pancreas β cells need to secrete more insulin to control blood sugar, and the body then appears hyperinsulinemic, revealed by a research consistent with the results of this study [18]. The above findings demonstrated that we successfully established a mice model of insulin resistance.

According to an investigation by previous literature, the development of insulin resistance is mostly associated with the ectopic deposition of lipids in the body and decompensated hyperplasia and hypertrophy of fat cells [19]. When lipid synthesis is increased, adipocytes undergo compensatory hyperplasia and hypertrophy to alleviate lipotoxicity in the circulatory system, and ectopic lipid deposition, until adipocytes decompensate in pathological states. Next, metabolic disorders of adipocytokines will trigger a cascade of signals to reduce insulin sensitivity in peripheral tissues [20]. Excessive intake of fat results in lipid deposition in adipose tissue, skeletal muscle, liver and ovary. Then, two signalling molecules, INSR IRS-1, and IRS-2 are selected to reflect the expression of insulin signalling in the liver, skeletal muscle and ovary. In this paper, the expression levels of INSR and IRS-1 mRNA decreased gradually in the two peripheral insulin target organs (liver and skeletal muscle) of mice in the HF group 0–12 weeks after a high-fat and high-sugar diet. On the contrary, the expression levels of INSR and IRS-1 mRNA gradually increased in ovarian tissue of mice in the HF group after 0–12 weeks of high fat and high glucose feeding.

The above results may have an important correlation with the occurrence and even the pathogenesis of PCOS [21]. Insulin signalling includes a finely regulated relay of intracellular signals, that mostly involves the phosphorylation and dephosphorylation of signalling molecules, which are initiated from insulin binding to the INSR [22]. The binding of insulin to the INSR induces tyrosine phosphorylation of the IRS, and then transduces signals through the downstream enzymes, such as PI3K and Akt2. When the body tissue cell-related protease receptor loses feedback to insulin stimulation, the secreted insulin cannot play a regulatory role in time, which will produce an imbalance of blood glucose homoeostasis and an IR state [23]. Akamine et al. demonstrated that systemic insulin resistance and hyperinsulinemia occurred together with impairment of the IRS/PI3K/AKT insulin signalling pathway in the ovary of high-fat diet-induced female obese rats [24]. Furthermore, insulin resistance prompted a marked increase in ovarian androgen. The binding of androgen to their receptors promotes follicle membrane cell hyperplasia, ovulation reduction, and ovarian polycystic changes [25]. Some PCOS patients exhibit increased phosphorylation of the serine residues of the IRS-1 molecule, thereby preventing the INSR from exerting its signal transduction function [26].

During the change in insulin resistance formation state, different tissues showed slightly different expression of different signal molecules simultaneously, and the expression of the same signal molecule in various tissues also changed with time [27]. These findings indicated that insulin resistance might be tissue-specific and time-dependent. In other words, the content of INSRs in the liver and skeletal muscle gradually decreased with the progress of the insulin resistance state, but the expression of INSRs in the ovarian tissue gradually increased with the progress of the insulin resistance state. It further suggested that insulin bound to more INSRs in the ovarian than in the liver and skeletal muscle in insulin resistance. There are many reports on this phenomenon, but the detailed mechanism remains unclear [28–31]. Through this experiment, we speculated that the expression level of INSR-related mRNAs and proteins in various tissues may be different; also, lipotoxicity may have different choices for pancreatic islet signalling pathways in the liver, skeletal muscle, ovary and other tissues. However, the specific mechanism or structure to regulate the insulin signalling pathway in various tissues needs further exploration.

Altogether, this study improved people’s understanding to the early insulin resistance and metabolic syndrome, provided insights into the underlying mechanism of the occurrence and development of type 2 diabetes, and contributed to preventing insulin resistance. Hence, this study can be used as a reference to develop more effective preventive measures for obesity, T2DM, PCOS and other metabolic syndromes. However, there are still some limitations in this study. Firstly, we only perform the experiments based on the animal model but do not recruit sufficient clinical samples to verify. Secondly, it remains unclear whether there are the same changes in obesity, T2DM, PCOS and other animal models. Besides, the molecular mechanism of the changes of the INSRs in different organs is not further explored experimentally. Therefore, future study requires more basic experiments and clinical research.

## Conclusion

The expression levels of INSR, IRS-1 and p-IRS-1 in the liver and skeletal muscle of HF group mice decrease gradually with the progress of insulin resistance, while those in the ovary organ present the opposite trend. This study improved our understanding of early insulin resistance and metabolic syndrome, but the related molecular mechanisms warrant further investigation.

## Materials and methods

### Experimental grouping and animal model establishment

**Figure 7.**
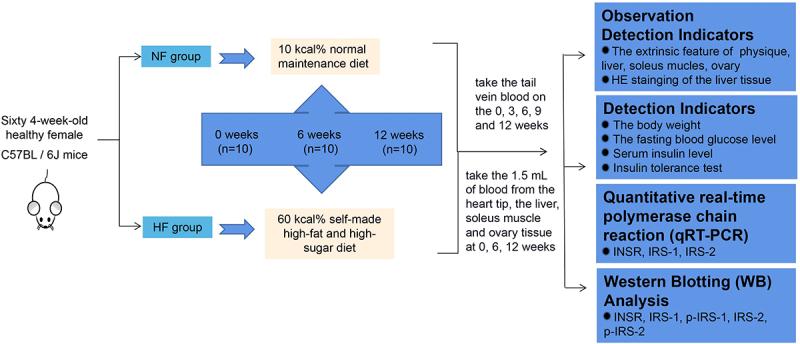
A schematic diagram of experimental design.

The animals used in this study were provided by SPF (Beijing) Biotechnology Co., Ltd. Sixty 4-week-old healthy female C57BL/6J mice with a body weight of 15–18 g were selected as the study objects. They were placed in a specific pathogen-free (SPF) animal feeding room with good ventilation and stable humidity, and had free access to food and water. After 2 weeks of adaptive feeding, the experiments were performed. Firstly, the mice were randomly divided into a normal diet group (NF group) and a high-fat and high-sugar diet group (HF group). After labelling, they were divided into 6 groups according to the random number table method, with 10 mice in each group. The first three groups belonged to the NF group, and the last three were in the HF group. The NF group and HF group were further divided into three groups according to the length of feeding time, namely, 0-, 6- and 12-week feeding groups. The mice in the NF group were fed with a normal maintenance diet with approximately 10 kcal%; mice in the HF group were given a self-made high-fat and high-sugar diet with approximately 60 kcal% (55% normal maintenance diet, calcium hydrogen phosphate 2.0%, soy protein isolate 10.0%, lard 12.0%, sucrose 20.0%, cholesterol 1.0%) [17,32]. The body weight of mice in each group was measured and recorded in the morning (12 h of fasting) after 0, 3, 6, 9 and 12 weeks of feeding. A schematic diagram of the experimental design was showed in Figure 7. This study was approved by the Ethics Committee of Ningxia Medical University (NYDDWLL20220021).

### Insulin tolerance test

After 12 weeks of feeding, the insulin tolerance test of mice was measured. Mice were intraperitoneally injected with 0.75 IU/kg of insulin, and their blood glucose was then measured at different time points. Next, the insulin tolerance curve was drawn, and the area under the curve (AUC) of ITT was calculated. At the end of the experiments, the mice were intraperitoneally injected with 50% glucose solution (2 mL/kg) to remove the hypoglycaemia state.

### Specimen collection

On the week 0, 3, 6, 9 and 12 of feeding, the tail vein blood of mice in each group was collected in the morning after 12 h of fasting to measure the FBG. At the end of different feeding periods, 10 mice in the NF and HF groups were taken out. Under anaesthesia, 1.5 mL of blood was collected from the heart tip and centrifuged at 3500 rpm/min at 4°C, 10 min later, the supernatant was stored at −20°C for subsequent detection of serum insulin. The liver, soleus muscle and ovary tissue of the mice were collected and photoed, then washed with PBS and placed in the −80°C refrigerator for standby.

### Serum and insulin detection

After fasting for 12 h, the tail vein blood of mice in each group was collected in the morning to measure the FBG level. Serum insulin levels were detected by an enzyme linked immunosorbent assay (ELISA). Besides, the homoeostasis model assessment parameter of insulin resistance (HOMA-IR) was calculated using the homoeostasis model (HOMA) to understand the degree of insulin resistance based on the following formula: HOMA-IR = (FBG × fasting plasma insulin [FINS])/22.5 [33].

### H&E staining

After 12 weeks of feeding, the liver tissues were washed with running water, dehydrated with alcohol, and transparentized with xylene. Then, the tissues were dewaxed, embedded, and then sectioned into 5 μm in thickness. After that, the tissue sections were stained using the H&E staining kit (C0105, Beyotime Biotechnology, China). In short, the sections were stained with haematoxylin for 5 min, differentiated with hydrochloric acid ethanol for 30 s, and then stained with eosin for 2 min. Next, they were dehydrated, transparentized and mounted. Finally, the pathological changes of liver tissues were observed under a microscope (CKX31, Olympus, Japan) at a magnification of × 200.

### Quantitative real-time polymerase chain reaction (qRT-PCR)

The total RNA was extracted from liver, skeletal muscle and ovarian tissues of mice according to Trizol RNA kit instructions (Thermo Fisher Scientific, Inc., Waltham, MA, USA). Next, cDNA was prepared by reverse transcription using the PrimeScript ™ RT Master Mix kit (TaKaRa, Osaka, Japan), and the target gene level was detected referring to SYBR GREEN kit instructions (Takara Biomedical Technology Co., Ltd., Osaka, Japan). Subsequently, polymerase chain reaction (PCR) reaction was completed on an Eppendorf gradient PCR instrument (Applied Biosystems, Foster City, CA). All primers were designed using the online primer 3 software and synthesized by Shanghai Sangong Bioengineering Co., Ltd. The forward primer sequences for INSR included 5’-AGGCTCCCGTCTCTTCTTCAA-3’, and the reverse was 5’-GACATCCCCACATTCCTCGTT-3’; the forward primer sequence for IRS-1 was 5’-GTTTCCAGAAGCAGCCAGAG-3’, and the reverse was 5’-ACTCTCTCCACCCAACGTGA-3’; the forward primer sequence for IRS-2 was 5’-CCCGAGTCAATAGCGGAGAC-3’, and the reverse was 5’-AGTGGCTCAGGGGTCTAT-3’; the forward primer sequence for the internal control β-Actin was 5’-TCACCCACACTGTGCCCATCTACGA-3’, and the reverse was 5’-GGATGCCACAGGATTCCATACCCA-3’. The reaction parameters were listed as follows: pre-denaturation at 95°C for 4 min, then 40 cycles of denaturation at 95°C for 10 s, annealing at 52°C for 30 s, and elongation at 72°C for 30 s, finally elongation at 72°C for 10 min. The Prism 7.0 software was used to map the CT value of each sample of qPCR using the 2 ^− ΔΔCt^ method.

### Western blotting (WB) analysis

The total protein was extracted from the liver, soleus muscle and ovary tissues of each group of mice, and the concentration of the sample protein was determined using the BCA kit (Beyotime, Shanghai, China). Then, 40 μg protein with 10 μL protein loading buffer was transferred to a 5 × sodium dodecyl sulphate-polyacrylamide gel electrophoresis (SDS-PAGE). When the bromophenol blue dye solution completely ran into the separation gel, the voltage was increased to 180 V until the bromophenol blue dye solution approached the lower edge of the gel. Then, the electrophoresis was stopped, the protein was transferred to a polyvinylidene fluoride (PVDF) membrane. The membrane was sealed with 5% skim milk powder for 1.5 h then incubated with corresponding primary antibodies (anti-β-actin, ab8226, 1:5000; INSR, ab283689, 1:1000; IRS-1, ab46800, 1:1000; p-IRS-1 (phospho Tyr632), ab109543, 1:1000; IRS-2, ab134101, 1:1000; and p-IRS-2 (phospho ser1149), ab178103, 1:1000, Abcam, Cambridge, UK) overnight at 4°C by shaking. The next day, the membrane was washed, then incubated with secondary antibody. Later, enhanced chemiluminescence (ECL) luminescent solution was applied to visualize the protein band in a dark room. Finally, Image J 180 software was employed to measure the grey value of each band of WB.

## Data statistics

Statistical analysis was performed through SPSS version 26.0 (SPSS Inc., Chicago, IL, USA). The data were expressed as means ± standard deviation (SD). Comparison among multiple groups was performed using one-way analysis of variance and the Tukey post hoc tes. A comparison between the two groups was conducted using the Student’s T-test. *P* < 0.05 indicates statistical significance.

## Data Availability

The data that support the findings of this study are openly available in ‘figshare’ at doi:10.6084/m9.figshare.22679746.
